# Advanced Imaging for Biopsy Guidance in Primary Brain Tumors

**DOI:** 10.7759/cureus.504

**Published:** 2016-02-22

**Authors:** Nelson Moussazadeh, Apostolos J Tsiouris, Rohan Ramakrishna

**Affiliations:** 1 Neurological Surgery, NewYork-Presbyterian/Weill Cornell Medical Center; 2 Radiology, Division of Neuroradiology, Weill Cornell Medical College; 3 Neurological Surgery, Weill Cornell Medical College

**Keywords:** magnetic resonance imaging, glioma surgery, perfusion imaging, gliomatosis cerebri, magnetic resonance spectroscopy, image-guided therapy

## Abstract

Accurate glioma sampling is required for diagnosis and establishing eligibility for relevant clinical trials. MR-based perfusion and spectroscopy sequences supplement conventional MR in noninvasively predicting the areas of highest tumor grade for biopsy. We report the case of a patient with gliomatosis cerebri and multifocal patchy enhancement in whom the combination of advanced and conventional imaging attributes successfully guided a diagnostic biopsy.

## Introduction

Brain tumors are imaged with magnetic resonance imaging (MRI), given its exquisite anatomic definition and diagnostic versatility. Many pathologies can be suggested on the basis of features seen on standard T1- and T2-weighted sequences alone, for example, diffuse intrinsic pontine glioma or juvenile pilocytic astrocytoma. Diffusion-weighted imaging demonstrates hypercellular and ischemic areas, with restricted Brownian motion correlating with a higher grade in gliomas [1]. Contrast enhancement indicates areas of high-grade disease with blood-brain barrier porosity. In focally enhancing and/or diffusion-restricting astrocytomas, a biopsy is targeted towards these areas as the highest histologic grade, in turn, guides treatment.

Supplemental MR perfusion imaging aids in tumor diagnosis, adding three to four minutes to the scanning time. In primary brain tumors, dynamic susceptibility contrast-enhanced relative cerebral blood volume (DSC rCBV) and dynamic contrast-enhanced plasma volume (DCE V_p_) have been used as tumor neovascularity surrogates. MR spectroscopy adds five to 10 minutes and noninvasively quantitates brain metabolites. N-acetyl aspartate (NAA; a neuron-specific molecule), choline (Cho; a cell membrane component reflective of cellularity), creatine (a metabolic surrogate), and lactate/lipid quantification (indicative of necrosis) improve upon standard diffusion and contrast mapping in predicting areas of low versus high (WHO II vs. III) histologic grade in astrocytoma [2-3]. In some cases, for example, where decreased choline differentiates contrast-enhancing radiation necrosis from the active tumor, spectroscopy can spare patients invasive sampling [4].

## Case presentation

### History and examination

A 57-year-old right-handed woman with hypertension and tobacco use presented with progressive word-finding difficulty. Neurologic examination was notable for mild anomic dysphasia, truncal ataxia upon tandem gait testing, and right-sided dysrhythmokinesis. She also reported nighttime headaches and memory loss. She was immunocompetent, afebrile, and had no clinical evidence of active infection or primary malignancy. 

### Imaging attributes

Non-contrast head CT revealed bilateral poorly defined intra-axial cerebral hemispheric lesions with vasogenic edema and scattered internal microcalcifications.

Gadolinium-enhanced brain MRI demonstrated predominately non-enhancing mass-like T2-hyperintense infiltrating lesions involving the left superior temporal gyrus, extending through the posterior left temporal and parietal lobes across the splenium of the corpus callosum into the right periatrial white matter, with additional noncontiguous signal abnormality in the left thalamus, corona radiata, and cortical regions in the left parietal and occipital lobes. In addition to the posterior left superior temporal gyrus, which was deemed inoperable, three other areas were diffusion-restricting: the medial left parietal lobe, left corona radiata and splenium of the corpus callosum, and the right periatrial white matter.

**Figure 1:**
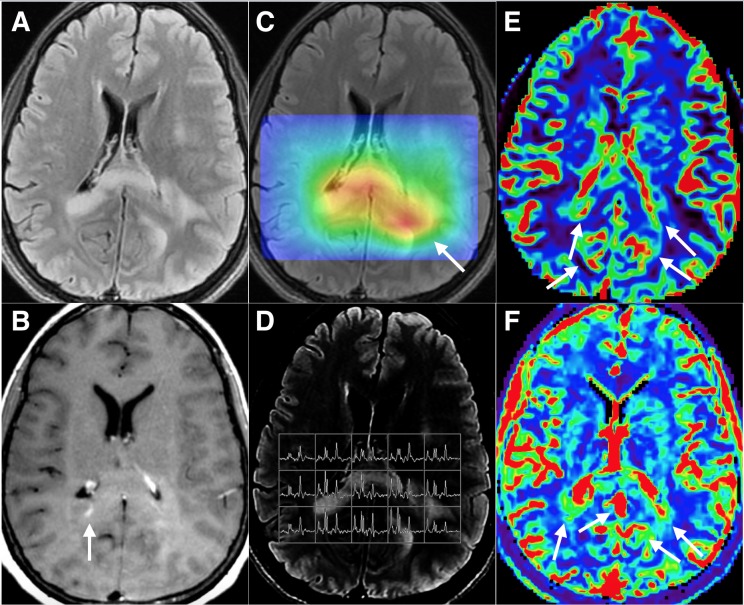
Advanced MR Characteristics A) Axial T2W-FLAIR demonstrates infiltrative disease in the right periatrial white matter, the bilateral forceps major, and deep left parietal lobe. B) Post-contrast T1W demonstrates a focus of enhancement in the right forceps major (arrow). C) Elevated choline (Cho):N-acetyl aspartate (NAA) ratios correspond well to the areas of T2 signal abnormality and represent neoplastic infiltration, best demonstrated on a color “heat map” generated from the multivoxel MR spectroscopy (GE AW READY View). D) Compared to the normal spectra (far right lateral voxels) with matched Cho and creatine (Cr) levels and high NAA levels, the infiltrated brain regions have a reversal of this pattern with elevated Cho and decreased NAA. E) The bilateral forceps major and splenium demonstrate multifocally increased cerebral blood volume (rCBV) on dynamic susceptibility contrast-enhanced (DSC) MRP (arrows). F) These same areas demonstrate increased plasma volume (V_p_) on dynamic contrast-enhanced (DCE) MRP (arrows). The left forceps major was chosen as the sampling site, despite not being avidly contrast-enhancing, as it was the area of highest Cho:NAA ratio, rCBV, and V_p_.

MR perfusion (MRP), spectroscopy (MRS), and post-contrast T1 imaging findings are presented in Figure 1.

Informed patient consent was obtained for this patient's treatment. No reference to the patient's identity was disclosed in the figure displayed in this paper.

## Discussion

This patient’s gliomatosis cerebri is unresectable, and the primary concern is establishing an accurate diagnosis via a minimally-invasive biopsy. Anatomic MR establishes this as an intra-axial tumor, most likely a glioma, given its confinement to and expansion of normal sulcal boundaries and diffuse infiltration of multiple lobes. Extension through multiple vascular territories and discontiguous gray and white matter involvement exclude ischemia and demyelination while viral encephalitis can be excluded clinically. By definition, this constitutes Type I gliomatosis cerebri as it diffusely involves ≥ 3 cerebral lobes [5]. Importantly, cell type and histologic grade guide gliomatosis therapy. While microcalcifications suggest oligodendroglial lineage, this and the tumor grade cannot be definitively assessed noninvasively. In combination with an ability to rule out atypical primary CNS lymphoma and cryptogenic metastasis, the goal of a biopsy is to safely sample the area of the highest predicted grade.

Standard contrast-enhanced T1- and diffusion-weighted sequences are typically adequate for biopsy planning. In this patient, where tissue adequacy for molecular profiling is critical, the only diffusion-restricting and contrast-enhancing area, which was in the right periatrial white matter, was small and in an area where a needle biopsy might return a non-diagnostic sample. However, elevated perfusion V_p_ and spectroscopic choline corroborated that area as the most likely to harbor high-grade features.

Additional advanced imaging modalities include CT/MR-fused positron-emission tomography (PET), a noninvasive adjunct examination for brain lesion diagnosis. Lacking the resolution of anatomic or perfusion MRI, PET may be useful in distinguishing radiation necrosis from an active tumor or biopsy planning in multifocal metastatic cases with unknown primary and sufficient spatial separation. While standard FDG-PET may be falsely negative and nonspecific for brain tumors, other amino acid tracers, including ^11^C-MET, have been shown to be more sensitive and specific in gliomatosis [6]. Diffusion tensor tractography may be informative in cases where normal eloquent fibers are suspected of traversing a lesion requiring biopsy.

This patient underwent navigation-assisted open biopsy with awake cortical language mapping to minimize the risk of postoperative dysphasia, given the proximity to Wernicke's area, and local sampling of the posterior left temporal lesion, which revealed anaplastic oligodendroglioma (WHO Grade III). Open biopsy was selected in this case over stereotactic biopsy given the small and deep target volume (< 0.5 cc^3) and the patient's desire for thorough tissue sampling and genetic sequence analysis for potential future clinical trial eligibility. Fluorescence in situ hybridization demonstrated 1p/19q codeletion (favorable), and immunohistochemistry was negative for IDH1 R132H mutation (wild-type is a negative prognostic) [7-8]. On the third postoperative day, the patient was discharged home with stable mild anomic dysphasia. At her outpatient visit three weeks later, she remained complication-free. However, breakthrough partial seizures required anticonvulsant titration, and she is planned for adjuvant whole brain radiation and procarbazine, lomustine, and vincristine therapy on the basis of RTOG 9402 and EORTC 26951 [9-10].

## Conclusions

Advanced MR-based imaging techniques may aid in glioma surgical planning. In gliomatosis cerebri, which is characterized by infiltration of ≥ 3 brain lobes, a stereotactic biopsy (or open biopsy, as clinically indicated) should be directed at the areas of predicted highest grade for treatment stratification. Lesional enhancement signifies breakdown of the blood-brain barrier, which is seen in tumors, but can also be seen in active demyelination, infection, and radiation necrosis, among other causes. Diffusion restriction is present in regions of tumor hypercellularity as well as infarction or purulence. The degree of Cho elevation and NAA depression on MR spectroscopy as well as plasma volume on DCE MRP, correlate with glioma grade.
